# The Relationship Between Activity Participation and Cognitive Functioning

**DOI:** 10.1093/geroni/igaa057.1371

**Published:** 2020-12-16

**Authors:** Sangha Jeon, Susan Charles

**Affiliations:** University of California-Irvine, Irvine, California, United States

## Abstract

Researchers are growing increasingly interested in how the diversity of daily activities are related to well-being. The current study examined how both frequency and diversity in daily activities are associated with cognitive functioning. Participants from the third wave (2013-2016) of the Midlife Development in the U.S (MIDUS) survey (N=1281) completed both a telephone-based cognitive assessment and a mailed survey asking about participation in three different types of activities: cognitive (e.g., doing word games, attending educational lectures or courses), physical (e.g,. exercise, home chores), and social (volunteer work, attending sports or social groups). Frequency of activity participation was assessed with items asking how often they engaged in these activities, and diversity of activity participation was calculated by summing the number of activities they reported doing in each category. All analyses included sociodemographic variables, health status, and openness to experience as covariates. Findings from multiple regression indicated that greater frequency in all activities (cognitive, physical, and social) was related to higher levels of cognitive functioning. Greater diversity of social activity was also related to higher cognitive functioning. Education mediated the association between diversity in cognitive activities and cognitive functioning, suggesting that the link between higher levels of cognitive functioning and education may be partly attributed to people with higher levels of education engaging in greater diversity of cognitive activity.

